# Colchicine versus cimetidine: the better choice for Periodic fever, aphthous stomatitis, pharyngitis, adenitis (PFAPA) syndrome prophylaxis, and the role of MEFV gene mutations

**DOI:** 10.1186/s12969-022-00733-3

**Published:** 2022-08-31

**Authors:** Seyed Reza Raeeskarami, Payman Sadeghi, Mahdieh Vahedi, Kosar Asna Ashari, Mahdieh Mousavi T., Vahid Ziaee

**Affiliations:** 1 Department Of Pediatrics, Imam Khomeini Hospital Complex, Tehran University Of Medical Sciences, Tehran, Iran; 2 Department of Pediatrics, Tehran University of Medical Sciences, Tehran, Iran; 3 Children’s Medical Center, Pediatrics Center of Excellence, Tehran, Iran; 4 Pediatric Rheumatology Research Group, Rheumatology Research Center, Tehran University of Medical Sciences, Tehran, Iran; 5 Clinical Research Development Unit of Akbar Hospital, Faculty of Medicine, Mashhad University of Medical Sciences, Mashhad, Iran; 6 Department of Pediatrics, Mashhad University of Medical Sciences, Mashhad, Iran; 7 Bahrami Children’s Hospital, Tehran, Iran

**Keywords:** Fever, Pharyngitis, Child, Colchicine, Cimetidine

## Abstract

**Background:**

During childhood, the most common periodic fever is periodic fever, aphthous stomatitis, pharyngitis, and cervical adenitis (PFAPA) syndrome. The effective treatment and prevention of febrile attacks improve these patients' and their families’ quality of life. However, there is no single strategy or evidence-based guideline to manage this syndrome, and most of them are based on consensus treatment plans.

**Methods:**

This randomized controlled trial was carried out on 67 PFAPA patients referred to three tertiary centers of pediatric rheumatology. The patients were divided into two groups, including group 1 (*n* = 36) receiving prednisolone plus colchicine and group 2 (*n* = 31) receiving prednisolone plus cimetidine. Demographic characteristics and the number of febrile episodes were compared between the two groups before and after the intervention.

**Results:**

In both groups, the number of febrile episodes after the treatment decreased (P ≤ 0.001). Statistical Analysis showed no significant difference between the two groups (*P* = 0.88). Moreover, 44 patients from both groups were checked for the MEFV gene. There were no statistical differences between MEFV positive and negative subgroups in response to colchicine (*P* = 1).

**Conclusion:**

This study showed that both drug regimens are significantly effective in preventing febrile attacks in PFAPA syndrome, and the presence of a MEFV gene mutation might not be the only significant risk factor for a response to colchicine.

**Trial registration:**

IRCT, IRCT20191222045847N1. Registered 23 October 2019, https://fa.irct.ir/search/result?query=IRCT20191222045847N1

## Background

Periodic fever, aphthous stomatitis, pharyngitis, and cervical adenitis (PFAPA) syndrome is the most common periodic fever syndrome in children, first described by Dr. Marshall et al. [1]. Fevers occur in an approximately clock-like manner every 3–8 weeks and usually last 3–5 days during some periods of the disease. Children have no symptoms or signs between the episodes of fever, and this syndrome does not affect normal growth and development [2]. Most of the time, these febrile attacks resolve spontaneously after childhood; However, there is a few risks of relapse in adulthood [3].

A new set of criteria was proposed in 2019, including mandatory criteria of a fever higher than 38.0 °C that lasts for less than 8 days (mean: 4 days) and recurs at least four times. At least five of eight supportive criteria are required to diagnose PFAPA, including the age of onset under 5 years, b) tonsillitis or pharyngitis with white moss, c) concomitant symptoms with at least one of the several clinical signs of aphthous stomatitis, cervical lymphadenitis, sore throat, vomiting, and severe headache, d) no cough, e) family history, f) inflammatory laboratory findings (e.g., C-reactive protein and serum amyloid A) that are remarkably raised during attacks but at normal levels in attack-free periods, g) high serum immunoglobulin D level, and h) glucocorticoid medication which is highly effective (Table 1) [4].

**Table 1:** Comparison of three classification criteria of PFAPA syndrome

classification criteria	Modified Marshall’s	Vanoni et al	Takeuchi et al
Fever	Regular recurrent fevers	Periodic Fever for at least 6 months: a. fever ≥ 38.5 °C b. Regular recurrent fevers ≥ 5 episodes, interval between fevers ≥ 2 months, Asymptomatic between episodes	Fever ≥ 38.0 °C that Recurs ≥ 4 times Asymptomatic between episodes
Duration of fever	-	2–7 days	< 8 day
Symptoms	Constitutional symptoms, absence of infection, ≥ 1 of the following signs: 1.Apthous stomatitis 2.Cervical lymphadenitis 3.Pharyngitis	Pharyngitis, cervical adenitis, Aphthous stomatitis, ≥ 1 in every episodes and ≥ 2 from 3 in the majority of episodes	Tonsillitis or pharyngitis Accompanying symptoms with ≥ 1 of the following signs 1.Aphthous stomatitis 2.cervical lymphadenitis 3.sore throat 4.vomiting 5.severe headache 6.No cough
Exclusion	Cyclic neutropenia	Other causes of recurrent fever	-
Growth and development	Normal Growth and development	Normal Growth	
Family history	-	-	A history of recurrent fever
laboratory Tests			Inflammatory findings (CRP & SAA) during febrile episodes Elevations of IgD
corticosteroids during febrile episode	-	-	Significant effect

*CRP* C‐reactive protein, *SAA* Serum amyloid, *IgD* Immunoglobulin D

Different medications have been recommended to treat and prevent febrile attacks. Treatment recommendations for PFAPA syndrome are mainly based on international consensus, physicians’ experience, and retrospective studies. The febrile attacks show a dramatic response to treatment with corticosteroids; nevertheless, there is a risk of increased frequency of the episodes in some patients [5]. There are a few clinical trials to evaluate cimetidine and colchicine effects [6, 7]. In most previous studies, the effects of prophylactic treatment with colchicine and cimetidine have been retrospectively investigated. Recommended treatments for preventing febrile attacks include colchicine, cimetidine, and tonsillectomy. Cimetidine has been used for PFAPA prophylaxis since 1992; nonetheless, it showed different efficacy among different populations [8]. Colchicine is another option for PFAPA prophylaxis since this syndrome has numerous similarities to familial Mediterranean fever, and multiple studies have demonstrated its effectiveness [9].

**Fig. 1:**
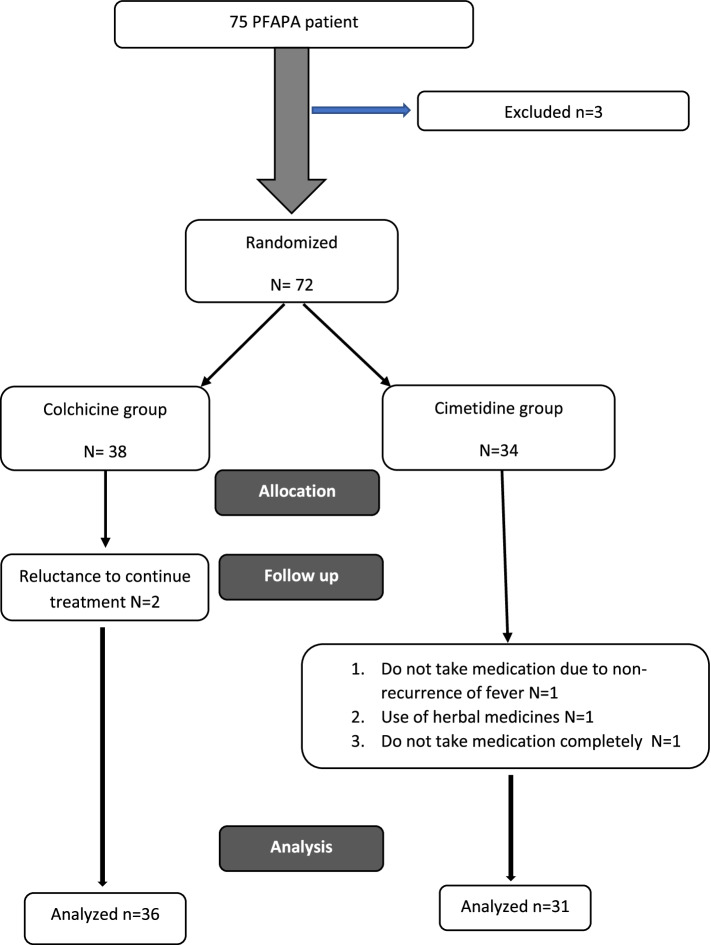
PRISMA flowchart of study

The primary purpose of this study was to compare the effectiveness of two accepted drugs, colchicine and cimetidine, in preventing febrile attacks of this disease (Fig. 1). Furthermore, this study evaluated the effect of *MEFV* gene mutation on patient’s response to colchicine. Therefore, this study investigated the role of the *MEFV* gene in influencing the choice of the treatment regimen as a sub goal.

## Methods

This study was performed as a randomized clinical trial on 67 children with PFAPA syndrome referred to the pediatric rheumatology clinic of Children’s Medical Center, Imam Khomeini hospital, and Bahrami Children’s hospital (all three centers affiliated with Tehran University of Medical Science, Tehran, Iran within 2019 to 2021 (before the coronavirus disease 2021 pandemic in Iran). In routine outpatient visits in our pediatric rheumatology centers, the diagnosis of PFAPA syndrome is made on Vanoni et al. criteria [10]. But all of the patients have been also diagnosed by a modified Marshall's criteria [11]. The exclusion criteria were 1) occurrence of any other rheumatic or autoinflammatory diseases during treatment, 2) changing the patient’s diagnosis during treatment based on clinical or laboratory findings, 3) lack of proper follow-up, and 4) use of other treatments that affect the course of the disease, such as montelukast, special hypoallergenic diets. Colchicine was administered based on body weight (under 15 kg: 0.25 mg; 15–30 kg: 0.5 mg; over 30 kg: 1 mg) and cimetidine at a dose of 20–40 mg/kg/day. Throughout the study period (before and after treatment with colchicine or cimetidine), all patients received prednisolone at the onset of a febrile attack. Therefore, the use of corticosteroids is not considered as a confounding factor. None of our patients underwent tonsillectomy before or after the study.

Sample size calculations were performed using G*Power software (version 1.03). According to Butbul et al. [6], the minimum sample size was determined to be 44 patients, 22 patients in each group, with the hypothesis that an average of 1.6 episodes of fever would occur in the colchicine group and 3 episodes of fever in the cimetidine group. After determining the sample size, the number of samples was expanded to increase the study’s accuracy. The error level was 5%, and the power confidence interval was 95%. A *p*-value less than 0.05 was considered statistically significant.The randomization process was performed using a sealed envelope online site (https://www.sealedenvelope.com/) by the block randomization method. This process was repeated for every patient. After randomization, the physician prescribed the drug and it was bought from the drugstore by the patient.

Response to treatment was compared between the two groups by comparing the number of febrile episodes and days without fever before and after the treatment. In our study patients were followed three months after starting the treatment to evaluate their response. Follow-up was on call and every patient visited during each attack to check if he or she meets the criteria or not. During these visits, the compliance to drug therapy was followed by the physician. No serious adverse effects of drugs were observed during the follow-up period.

All the information was documented on the questionnaire forms which were designed for the study. Data analysis was performed using SPSS statistical software (version 26). Datasets were compared between the two groups using Pearson’s Chi-squared test, Fisher’s exact test, and T-test (i.e., independent samples test). In each group, the changes in the number of febrile attacks were assessed before and after the treatment using the Wilcoxon signed-rank test. The number of febrile attacks before and after the treatment was also compared between the two groups by repeated measures analysis.

## Results

In this study, 67 PFAPA patients were randomly divided into two groups and received colchicine or cimetidine as prophylaxis for their disease. In this study, 48 (69%) and 21 (31%) patients were male and female, respectively. In 13 patients, the parents had a consanguineous relationship. Table 2 shows a summary of the demographic characteristics of two groups. There was no statistically significant difference between the two groups in these characteristics.

**Table 2:** Demographic data and other characteristics of patients

Characteristics	Colchicine group	Cimetidine group	*P*-value
**Age (months)(mean ± SD)**	73.22 ± 28.47	76.16 ± 29.44	0.68
**Age at onset (months) (mean ± SD)**	27.19 ± 19.77	28.42 ± 23.19	0.81
**Sex**			
Female	12 (33%)	9 (29%)	0.70
Male	24 (67%)	22 (71%)	
**Weight (Kg)**	21.88 ± 6.73	21.59 ± 6.16	0.85
**Positive family history**^a^	4(11%)	3(10%)	0.58
**Parents blood relatives**	8(22%)	5(16%)	0.52

^a^Positive family history of recurrent fevers

Table 3 shows the clinical manifestations of the two groups. Again, there was no significant statistical difference between the two groups.

**Table 3:** Clinical symptoms and signs of the patients in two groups

Variable	Colchicine group	Cimetidine group	Total	*P*-Value
Fever	36(100%)	31(100%)	67	**0.76**
Pharyngitis	33(92%)	27 (87%)	60 (90%)	**0.69**
Oral Aphthous	30(83%)	24(77%)	54 (81%)	**0.54**
Lymphadenopathy	27(75%)	20(65%)	47 (70%)	**0.35**
Abdominal pain	25(69%)	16(52%)	41 (61%)	**0.13**
Arthralgia	17(47%)	16(52%)	33 (49%)	**0.72**
Headache	11(31%)	9(29%)	20 (30%)	**0.89**
Rash	3(8%)	0(0%)	3 (5%)	**0.24**

The trend of changes in the number of febrile attacks in patients was evaluated before and after the treatment that showed a significant decrease in both groups by repeated measures analysis. The number of attacks (in general) decreased significantly after the treatment, with no significant difference in efficacy (Table 4).

**Table 4:** Number of febrile attacks before and after treatment in two groups

**variable**	**Drug regimens**	**Mean ± SD**		***P* value**^a^	***P* value**^b^
		Three months before treatment	Three months after treatment		
**febrile attacks (number)**	Colchicine	5.11 ± 2.03	2.17 ± 1.79	≤ 0.0001	0.88
	Cimetidine	4.65 ± 2.15	2.52 ± 1.76	≤ 0.0001	

^a^*P* within Subjects Effects/ Repeated Measures analysis

^b^*P* between Subjects Effects/ Repeated Measures analysis

Laboratory studies were done at the beginning of the study to rule out other diagnoses. Acute-phase reactants, including white blood cells during febrile attacks, were compared between the two groups by independent samples test, and there was no difference between the two groups in white blood cell count and erythrocyte sedimentation rate (Table 5).

**Table 5:** Laboratory examination during febrile episodes in patients

**variable**	Mean ± SD		
	Colchicine group	Cimetidine group	***P*-value**
**White Blood Cell /ml**	12,719.34 ± 4.4	12,725.39 ± 8.6	0.99
**Platelets /mcl**	322,427.92 ± 7.9	274,709.96501 ± 6.5	0.04
**Erythrocyte Sedimentation Rate (ESR) mm/hr**	31.18 ± 5.8	33.18 ± 9.5	0.59
**C-Reactive Protein mg/dl**	41.14 ± 9.8	34.11 ± 2.9	0.02

MEFV genes were examined in 44 of 67 patients, and mutations were observed in 16 of 44 (36%) patients. There was a MEFV mutation in 11 and 5 patients in the colchicine and cimetidine groups, respectively. There was no statistical differences between two groups. (*P* = 0.32). The most common MEFV mutation was E148Q. Moreover, 13 (81%) and 2 (13%) patients had heterozygous E148Q variant and heterozygous P369S/R480Q mutation in the MEFV gene, respectively. One of the patients was a heterozygous carrier of R761H mutation in the MEFV gene.

For the evaluation of the effect of the MEFV gene on the patient’s responses, these groups of patients were divided into two subgroups, namely responders (reducing the number of febrile attacks ≥ 40%) and non-responders. The frequency of MEFV gene mutation was compared between the two groups by the Fisher’s exact test, which revealed no statistically significant difference (*P* = 1) (Table 6).

**Table 6:** Frequency of MEFV gene mutations in responder group and non-responder group

	**variable**		**Responder group**	**Nonresponder group**	**total**	*P-value
**MEFV mutation**	**Cimetidine**	**Carriers (number)**	4 (36%)	1 (14%)	5 (28%)	1
		**Non Carriers (number)**	7(64%)	6 (86%)	13 (72%)	
	**colchicine**	**Carriers (number)**	7(41%)	4(44%)	11(42%)	1
		**Non Carriers (number)**	10(59%)	5(56%)	15(58%)	

^*^Fisher’s Exact Test

## Discussion

Although PFAPA syndrome is a benign autoinflammatory disorder, Serious complications such as amyloidosis in PFAPA syndrome are very rare. Since this condition can affect the patient’s quality of life and have a profound impact on family economy, decision about choosing the better option to prevent of febrile episodes is one of the therapeutic challenges [12]. Recommended treatments for prophylaxis of febrile attacks are based on retrospective studies and the international consensus of physicians [13–15]. There are few randomized controlled trials of the prophylactic treatment in PFAPA syndrome. This study was a randomized clinical trial to evaluate and compare the effect of colchicine and cimetidine in preventing febrile episodes.

In the present study, the male/female ratio was about 2/1, and the age of disease onset was within 6 months to 4.5 years, which is nearly consistent with the results of previous studies in Turkey and Slovenia. In most previous studies a male significant predominance in the incidence of PFAPA syndrome has been reported [16, 17]. There was no significant difference in demographic characteristics between the colchicine and cimetidine groups, as shown in Table 2. There was a positive family history of recurrent fever in first-degree relatives in 7 (10%) patients. Moreover, 13 (19%) patients had parents with consanguineous marriages. A family history of recurrent fever was reported within 10–44% in other studies. This finding could suggest the genetic background of PFAPA syndrome [18–20]. Further investigations of genetic causes in PFAPA syndrome help diagnose the etiology of this disease.

Table 3 shows the frequency of different clinical symptoms and signs. Febrile attacks are mandatory for diagnosis, and pharyngitis was the most common symptom, as in numerous other studies. Abdominal pain was more common among patients in this study. There was higher incidence of abdominal pain in our patients which could be due to the high incidence of MEFV mutations in our population compared to other countries. We can't generalize the results to other populations with a low incidence of MEFV gene mutations [4, 17, 21]. Also, the high mean number of attacks before treatment could be attributed to seeking more for prophylactic therapy among patients with more active disease.

Cimetidine is one of the first drugs introduced for PFAPA prophylaxis [8]. This drug is an H2 blocker with immune-modulating properties, especially on T cells. Cimetidine efficacy has been reported within 25–43% using 10–20 mg/kg twice daily. Thomas et al. and Wurster et al. recorded the effect of this drug on the prevention of febrile attacks in some PFAPA patients by studying their medical records [2, 22, 23]. Cimetidine has also been used in early infancy to control febrile attacks and postpone more invasive therapies, such as tonsillectomy [24]. According to our review of the past literature, it seems there is no clinical trial that evaluates the effect of cimetidine in the prevention of febrile attacks in the PFAPF syndrome. In most previous studies, the effects of prophylactic treatment with colchicine and cimetidine have been retrospectively investigated.

In studies on the patients of the Eurofever registry or the Norwegian cohort, no patient was treated with cimetidine [12]. Therefore, the current study has been one of the first trials that could show the efficacy of cimetidine in PFAPA prophylaxis by significantly reducing the number of febrile attacks.

Colchicine was also tried to prevent the attacks due to the cyclic pattern of fevers in PFAPA and its resemblance to other hereditary autoinflammatory diseases, especially familial Mediterranean fever [25]. Colchicine can even be used in patients with a clinical diagnosis of autoinflammatory diseases in the absence of pathogenic gene variants [26]. The mechanism of action of colchicine in reducing inflammation is unknown. Colchicine binds to tubulin and forms the colchicine tubulin complex. This complex can alter the structure and function of the cytoskeleton and affect the migration of neutrophils and lymphocytes [7].

PFAPA syndrome is more common in the Mediterranean race. The rate of MEFV gene mutations in patients with PFAPA syndrome is also higher than in the general population.

According to previous studies, PFAPA patients with MEFV gene mutations have a better response to colchicine. Therefore, it seems that patients of the Mediterranean race respond better to treatment with colchicine [27–29]. This study couldn't approve this hypothesis. The main reason could be due to mixed ethnicity in our patients and the high prevalence of less important mutations [30].

Welzel et al. concluded that colchicine is effective in PFAPA even when there is no *MEFV* mutation, which is consistent with the results of the present study [9]. It could increase the time interval or complete remission. Gunes et al. reported that in patients with PFAPA syndrome and *MEFV* gene mutations, colchicine is more effective as a prophylactic treatment. In the current study, the prevalence of *MEFV* mutation in the responders to colchicine and non-responders showed no significant difference [7].

Types of MEFV gene mutations are important in therapeutic response.In studies that emphasize the effect of colchicine on patients with PFAPA syndrome who are carriers of the *MEFV* mutation, the most commonly reported mutation in ethnic groups such as the Turkish people was the M694V [31]. It is one of the primary mutations in the *MEFV* gene with high penetrance. In the current study, there was no statistically significant difference between the responder and non-responder groups in any prophylaxis treatment in the presence or absence of *MEFV* mutation. Since the most common mutation was E148Q in the present study, the discrepancy between the results of the current study and previous studies could be explained by differences in the prevalence of the mutation type and its penetrance.

## Conclusion

Cimetidine’s prophylactic effect in PFAPA syndrome has received less attention from physicians in recent years, and colchicine’s effect has not been confirmed by randomized clinical trials. The present study showed that both colchicine and cimetidine regimens are effective in reducing the number of febrile episodes and increasing the number of days without fever. There was no statistically significant difference between the two groups. Therefore, treatment with both regimens can be used to prevent fever attacks. In the current study, the frequency of *MEFV* gene mutations was not higher in the responder group; therefore, there might be other mechanisms involved in the pathogenesis of PFAPA syndrome in addition to *MEFV* gene mutation.

### Advantages and limitations

The main challenge tried to respond to was choosing a better PFAPA prophylaxis option. To the best of our knowledge, this study has been one of the first studies comparing the effects of cimetidine and colchicine on the prevention of febrile episodes in PFAPA syndrome in a clinical trial. According to the small number of clinical trial studies related to effective therapies in preventing febrile episodes in PFAPA syndrome, it is recommended to conduct multiple randomized prospective clinical trial studies with large sample sizes and racially diverse populations.

It was impossible to study *MEFV* gene mutation in all patients due to the high cost of the test. In future studies, it is best to evaluate *MEFV* mutation in all patients. Due to the presence of a family history of recurrent fever in patients and a high frequency of parents with consanguineous marriages, further studies are recommended to evaluate the possible genetic causes of PFAPA syndrome.

There was higher incidence of abdominal pain in this clinical trial which could be due to the high incidence of MEFV mutations in our population compared to other countries. Since we didn't collect any data about the pattern and severity of abdominal pain, we can't generalize this result to other populations with low incidence of MEFV gene mutations.

## Data Availability

The datasets used and/or analyzed during the current study are available from the corresponding authors on reasonable request.

## Abbreviations

PFAPA: Periodic Fever, Aphthous Stomatitis, Pharyngitis, Adenitis
CTP: Consensus Treatment Plans
RCT: Randomized controlled trial
MEFV gene: Mediterranean Fever gene
